# Comparison of two CT-based methods for tibial bone mineral density assessment and their associations with bone and eggshell traits in laying hens

**DOI:** 10.3389/fvets.2025.1709810

**Published:** 2025-12-15

**Authors:** Webert Aurino da Silva, Carlos Bôa-Viagem Rabello, Lilian Francisco Arantes de Souza, Elton Francisco de Oliveira, Adrielle Carneiro Araújo Santos, Leanndra de Pádua Ferreira Santos, Apolonio Gomes Ribeiro, Júlio Cézar dos Santos Nascimento, Marcos José Batista dos Santos, Lucas Rannier Ribeiro Antonino Carvalho, Fabiano Sellos Costa

**Affiliations:** 1 Department of Animal Science, Federal Rural University of Pernambuco, Recife, Brazil; 2 Department of Animal Science, Federal University of Paraiba, Areia, Brazil; 3 Department of Physiology and Pharmacology, Karolinska Institutet, Stockholm, Sweden; 4 Department of Veterinary Medicine, Federal Rural University of Pernambuco, Recife, Brazil

**Keywords:** bone strength, quantitative imaging, skeletal integrity, eggshell quality, bone mineralization

## Abstract

**Introduction:**

Bone quality is essential for the health, welfare, and productivity of laying hens. Quantitative computed tomography (QCT) allows accurate evaluation of bone mineral density (BMD), but methodological differences may influence the reliability of results. This study compared two QCT-based methodologies for tibial BMD assessment and investigated their associations with bone composition and eggshell quality traits.

**Methods:**

Forty-eight 48-week-old Dekalb White hens were evaluated. Method 1 (M1) measured BMD in four predefined cortical quadrants used as regions of interest. Method 2 (M2) applied semi-automatic segmentation of the entire bone area using predefined density thresholds. BMD results were compared between methods, and correlations were calculated with bone composition (bone weight and volume) and eggshell quality (shell weight, thickness, and breaking strength).

**Results:**

The methods showed moderate correlation (*r* = 0.6822, *p* < 0.001) but low concordance (CCC = 0.120), with M1 consistently overestimating BMD. Mean BMD values were 1152.49 ± 218.54 mgHA/cm^3^ for M1 and 711.22 ± 118.40 mgHA/cm^3^ for M2. M1 demonstrated weak correlations with bone parameters (bone weight: R^2^ = 0.423, *p* = 0.038; bone volume: R^2^ = 0.086, *p* = 0.043) and minimal associations with eggshell traits. In contrast, M2 showed stronger relationships with bone composition (bone weight: R^2^ = 0.789, *p* = 0.003; bone volume: R^2^ = 0.535, *p* = 0.010) and significant negative correlations with eggshell weight (R^2^ = –0.741, *p* = 0.009), thickness (R^2^ = –0.617, *p* = 0.017), and breaking strength (R^2^ = –0.654, *p* = 0.048).

**Discussion:**

M2 provided more accurate, consistent, and biologically meaningful BMD estimates than M1. Its stronger associations with bone and eggshell parameters support the adoption of M2 as a reliable QCT-based methodology for evaluating bone quality in laying hens.

## 1 Introduction

The laying hen industry, responsible for global egg production, has undergone a profound intensification process in recent decades, resulting in substantial gains in productivity per bird (1). These advances are mainly attributed to genetic improvement, precision nutrition, and enhanced management practices (2). However, the pursuit of high productivity levels has raised significant concerns regarding the physiological health and welfare of laying hens, as the intense and continuous egg-laying process imposes substantial metabolic demands that lead to a gradual deterioration of overall health (3).

Particularly, there is a deterioration of skeletal integrity with hen age, as large amounts of calcium (approximately 2 g per day) are exported for eggshell formation, coming partly from the diet and partly from the skeleton, mainly from medullary bone. Since production intensification may increase the risk of bone fractures and behavioral restrictions, particularly in conventional cage systems (4, 5).

In this context, bone quality in laying hens is a key factor both for animal welfare and for sustaining egg production (6). Eggshell formation requires a large amount of calcium, mobilized mainly from medullary bone during the night and replenished by dietary absorption in the morning to sustain continuous shell production (7). This process compromises skeletal integrity and may lead to osteoporosis and fractures, particularly in the keel bone and tibia, where callus formation increases density in the former and bone loss reduces it in the latter (8).

Studies have shown that genetic, nutritional, and management strategies can reconcile high egg production with adequate bone quality, with bone mineral density (BMD) serving as a central parameter to evaluate skeletal integrity and overall bone health (9–11). Higher BMD values are associated with a lower incidence of deformities and fractures, thereby contributing to productive performance and reducing economic losses, whereas a reduction in BMD has been linked to eggs with thicker and stronger shells, suggesting a compensatory redistribution of calcium toward eggshell formation (12, 13).

Accordingly, tibial BMD assessment is used to understand bone strength, mineralization, and the effects of genetic, nutritional, and environmental factors on skeletal integrity (14–17). Several methodologies are available, including destructive techniques, such as ash analysis, and non-destructive methods, such as dual-energy X-ray absorptiometry (DEXA), quantitative ultrasound (QUS), and quantitative computed tomography (QCT) (18–20). QCT, due to its accuracy and non-invasive nature, has emerged as a valuable tool for measuring BMD in poultry, gradually replacing traditional destructive methods (21, 22).

Nevertheless, there is still no standardized approach for analyzing tomographic images. Some studies rely on restricted cortical regions, such as quadrant-based analyses (23, 24), whereas Harrison et al. (25), advocates for whole-area segmentation of the region of interest.

This lack of methodological uniformity hinders comparability across studies and limits progress in this field. Considering this gap, the present study aimed to compare two QCT-based methodologies for assessing tibial bone mineral density (BMD) in laying hens and to evaluate their correlations with bone composition and eggshell quality. These analyses were conducted to identify the most consistent and biologically representative approach for studying bone quality and to provide insights into skeletal health in commercial egg production.

## 2 Materials and methods

### 2.1 Study site, animals, and housing conditions

The experiment was conducted at the Poultry Research Laboratory (LAPAVE) of the Department of Animal Science, Federal Rural University of Pernambuco (UFRPE), Recife, PE, Brazil (Latitude: 8°01′11.3”S; Longitude: 34°57′14.6”W). The entire experimental protocol was approved by the Institutional Animal Care and Use Committee (CEUA) under approval number 8680290224.

A total of 48 commercial Dekalb White laying hens, 48 weeks of age, were used. The birds had an average body weight of 1,575 ± 125 g and were housed in conventional non-furnished cages equipped with trough feeders and nipple drinkers. Water was supplied *ad libitum*, and feed was formulated to meet the nutritional requirements for the production phase, according to the breeder’s management guide.

### 2.2 Euthanasia, sample collection, and preparation

Birds were euthanized by cervical dislocation following a two-step protocol. First, they were induced into deep anesthesia via intravenous administration of propofol, titrated to the loss of reflexes. Euthanasia was performed only after complete unconsciousness was confirmed.

After euthanasia, the right tibia of each bird was collected, dissected, and carefully cleaned of all adhering soft tissues using scalpel blades to preserve bone integrity. Prior to imaging, the bones were thawed under refrigeration (4 °C) for 24 h and then maintained at room temperature for 2 h to ensure thermal equilibration. Each sample was then scanned by computed tomography (CT) to determine bone mineral density (BMD). The same bones were subsequently used for composition analyses, including weight, length, mineral matter, volume, Seedor index and ash content.

### 2.3 Computed tomography (QCT) image acquisition

Computed tomography scans were performed using a multislice Bright-Speed scanner (General Electric, Fairfield, CT 06824, USA), calibrated prior to each session. Tibiae were positioned in a standardized manner, oriented from proximal to distal, to ensure stability and uniform alignment during scanning along the longitudinal axis.

Transverse slices of 1 mm thickness were obtained at 1 mm intervals. Acquisition parameters included a tube rotation time of 1 s, 120 kV, and automatic tube current (mA). Images were reconstructed using a bone window.

For density calibration, a QCT phantom (IA® Image Analysis, Inc., US patents 4,233,507 & 4,922,915) was placed ventrally to the tibiae during each scan. The phantom contained reference cylinders with known calcium hydroxyapatite concentrations (0, 100, and 200 (mgHA/cm^3^)), enabling the conversion of Hounsfield Units (HU) to bone mineral density in mgHA/cm^3^.

### 2.4 Image analysis and methodological comparison for bone density assessment

Tomographic images were evaluated using DICOM viewing software (Horos, Purview, Annapolis, MD, USA; version 3.3.5) on a workstation (Apple MacBook Air, Apple).

**Figure 1:**
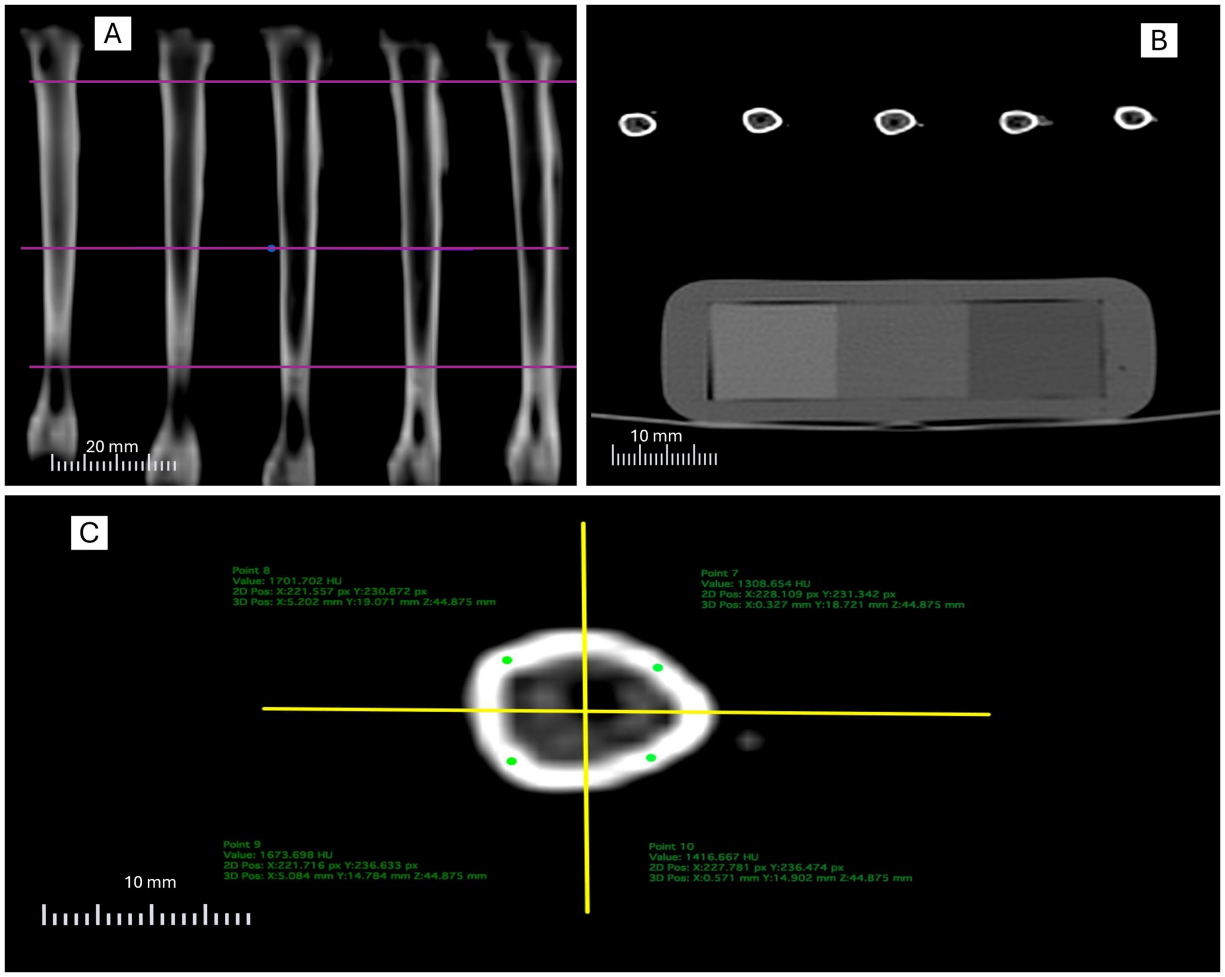
Illustration of the computed tomography analysis protocol: **(A)** definition of transverse assessment levels (proximal, medial, and distal); **(B)** positioning of the calibration phantom within the acquisition field; **(C)** division of the slice into anatomical quadrants and placement of regions of interest (ROI) in the cortical bone.

Transverse slices were standardized at three regions of the tibial diaphysis from all 48 tibiae (Figure 1A), corresponding to 25% (proximal), 50% (midshaft), and 75% (distal) of the total bone length, measured from the proximal end. In each slice, three replicates per region of interest (ROI) were obtained, and mean values were subsequently calculated according to the adopted methodology, resulting in 432 measurements per method. The within-method reproducibility was assessed by calculating the coefficient of variation (CV%) among the three replicates for each region. The mean CV was below 5% for both methods, indicating high precision and minimal non-biological variation in the CT measurements.

Cortical bone density was then assessed using two distinct methodologies to determine the most consistent approach.

#### 2.4.1 Method 1 (M1): quadrant-based analysis

Initially, tibiae were positioned in the scanner, and longitudinal plane images were acquired to define the transverse slices of interest (Figure 1A). Transverse slices were then obtained with the calibration phantom positioned within the acquisition field, ensuring standardization of radiodensity values and allowing correction of potential equipment-related variations (Figure 1B).

For cortical bone analysis, each transverse slice was virtually divided into four anatomical quadrants (anterior, posterior, medial, and lateral) using orthogonal axes drawn from the geometric center of the section. Within each quadrant, a circular region of interest (ROI) was placed in the cortical bone, prioritizing homogeneous areas with the highest radiodensity (mgHA/cm^3^) (Figure 1C). The mean BMD values from the ROIs were considered representative of each slice.

#### 2.4.2 Method 2 (M2): whole-bone segmentation (cortical + trabecular regions) analysis with density threshold segmentation

**Figure 2:**
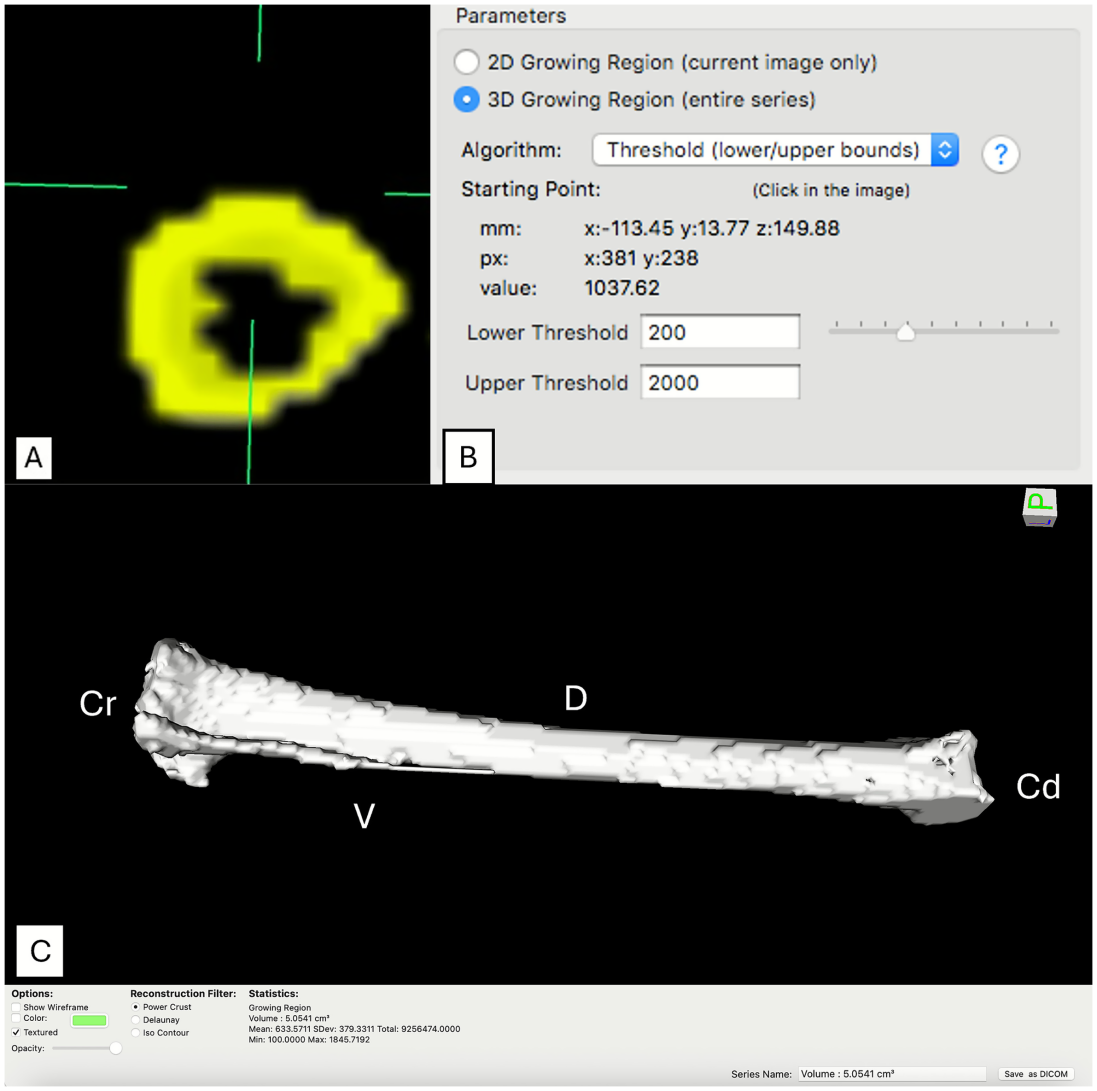
Segmentation procedure and cortical analysis by computed tomography. **(A)** Example of Region Growing segmentation using the “2D Grow Region” tool in a transverse tibial slice, in which the entire mineralized bone area (highlighted in yellow) was selected for evaluation; **(B)** definition of radiodensity threshold parameters (200 to 2000 BMD) for selective inclusion of cortical bone tissue; **(C)** three-dimensional reconstruction of the right tibia of laying hens obtained by computed tomography (CT). Anatomical orientation is indicated (Cr, cranial; Cd, caudal; D, dorsal; V, ventral).

In this second methodology, the entire bone of each slice (proximal, medial, and distal)—the same regions analyzed in M1—was defined as the ROI. Selection was performed semi-automatically through the following steps:

1. *Region Growing Segmentation*: The “2D Grow Region” tool was applied, an algorithm that expands the selection from a seed point to include all adjacent pixels within a predefined density range (Figure 2A).
2. *Density Threshold Definition*: Algorithm parameters were set with a lower threshold of 200 BMD and an upper threshold of 2000 BMD. This range was chosen based on the HU radiodensity scale, a universal standard in CT imaging (Figure 2B). The lower limit of 200 BMD was adopted to effectively distinguish bone tissue from surrounding soft tissues, since muscle and other soft tissues typically present lower radiodensities (26). The upper limit of 2000 BMD was defined to encompass the broad range of cortical bone density while minimizing the inclusion of hyperdense artifacts, such as those arising from metallic materials.
3. *Mean Radiodensity Calculation*: After precise ROI selection encompassing the entire cortical bone, the software automatically calculated the mean radiodensity (HU) of the delimited region. Additionally, using the “ROI volume” tool, volumetric metrics were obtained, including bone volume, mean radiodensity, minimum and maximum values, standard deviation, and three-dimensional (3D) reconstruction of the tibia (Figure 2C).

### 2.5 Calculation of bone mineral density (BMD)

For both methodologies, the radiodensity obtained (HUt) was converted into BMD, expressed in mg/cm^3^ of calcium hydroxyapatite, using the linear calibration equation derived from the reference phantoms scanned together with the samples:


BMD(mg/cm3)=200×HUt/(HUb−HUw)

Where:

HUt is the radiodensity of the tibial bone.

HUb is the radiodensity of the bone phantom (reference of 200 mgHA/cm^3^ of hydroxyapatite).

HUw is the radiodensity of the water phantom (reference of 0 mgHA/cm^3^).

This approach has been previously described (27–29). Measurements were initially obtained from three standardized tibial regions (proximal, medial, and distal). Subsequently, regional values were pooled and averaged to generate a representative whole-bone value for each methodology. This strategy provided greater statistical robustness and enabled direct comparison between methodologies.

### 2.6 Bone parameters and eggshell quality assessment

Following the tomographic analyses, the same tibiae were used to determine their physical properties and composition. Bone length (mm) was measured from the proximal to the distal epiphysis using a precision digital caliper. Bone weight (g) was recorded on an analytical balance (0.001 g). For mineral matter determination, bones were dried in a forced-air oven at 105 °C for 72 h to obtain dry weight. Subsequently, they were incinerated in a muffle furnace at 600 °C for 24 h to quantify mineral matter (ash), expressed in grams (g) and as a percentage (%) of bone-dry weight. The Seedor index was calculated by dividing bone weight (mg) by bone length (mm) (30).

In parallel, for eggshell quality assessment, three eggs from each hen were collected over three consecutive days preceding euthanasia (9 eggs per bird). Eggshell breaking strength (N) was determined at the equatorial region using a digital egg tester (Model DET6500, Nabel Co., Ltd.®, Kyoto, Japan). After testing, eggshells were washed, air-dried for 48 h, and weighed to determine shell weight (g). Shell thickness (mm) was obtained as the means of three measurements taken at different points on each egg using a digital micrometer.

### 2.7 Statistical analyses

Analyses were performed in R Studio, version 4.5.1. Agreement between BMD assessment methods, M1 and M2, was evaluated using Pearson correlation, Bland–Altman plots (31), Concordance Correlation Coefficient (CCC), and Deming regression, thereby quantifying linear association, bias, precision, and accuracy in an integrated manner. All analyses were conducted using BMD values expressed in milligrams of hydroxyapatite per cubic centimeter (mgHA/cm^3^), which were obtained from the calibrated CT data. Prior to analysis, data were tested for normality using the Shapiro–Wilk test, and assumptions of homoscedasticity and linearity were verified.

Precision of each method was also evaluated through the coefficient of variation (CV) and confidence intervals (CI), providing measures of variability and reproducibility of BMD measurements for M1 and M2.

To minimize the effect of bone size, BMD values were normalized by bone volume and correlated with bone parameters (length, mineral mass, Seedor index, bone volume) and eggshell quality traits (weight, thickness, and strength) using simple linear regressions. The coefficient of determination (R^2^) was used to indicate the proportion of explained variation, with a significance level set at 5% (*p* < 0.05), allowing for comparison of the explanatory power of M1 and M2 regarding bone properties and eggshell quality in laying hens.

## 3 Results

### 3.1 Agreement between methods (M1 vs. M2)

A total of 48 samples were analyzed using each bone mineral density (BMD) assessment method. Method 1 (M1) showed a mean of 1152.49 mgHA/cm^3^, with a standard deviation of 218.54 and a coefficient of variation (CV) of 18.96%. Method 2 (M2) yielded a mean of 711.22 BMD, with a standard deviation of 118.45 and a CV of 16.65% (Table 1).

**Table 1:** Descriptive statistics of the two methodologies for bone mineral density (BMD) assessment.

Descriptive statistics	M1	M2
Sample size (*n*)	48	48
Mean BMD (mgHA/cm^3^)	1152.49	711.22
Standard deviation	218.54	118.45
Coefficient of variation (%)	18.96	16.65

Regional bone mineral density (BMD) values for the tibia are summarized in Table 2. In both CT-based methods, BMD varied along the tibial diaphysis, showing the highest mean values in the medial region, followed by distal and proximal portions. For M1, BMD ranged from 1120.17 ± 205.18 mgHA/cm^3^ in the proximal segment to 1185.19 ± 230.22 mgHA/cm^3^ in the medial region, representing an increase of approximately 5.5%. For M2, BMD ranged from 689.91 ± 115.09 mgHA/cm^3^ to 729.87 ± 120.14 mgHA/cm^3^, showing a similar difference of about 5.5% between the same regions. The coefficients of variation ranged from 18.33 to 19.42% for M1 and 16.42 to 16.82% for M2.

**Table 2:** Mean (±SD) bone mineral density (BMD mgHA/cm^3^) per tibial region for the two CT-based methods.

Tibial region	M1 Mean (mgHA/cm^3^) ± SD	CV (%)	M2 Mean (mgHA/cm^3^) ± SD	CV (%)
Proximal	1120.17 ± 205.18	18.33	689.91 ± 115.09	16.71
Medial	1185.19 ± 230.22	19.42	729.87 ± 120.14	16.42
Distal	1152.12 ± 220.23	19.13	713.89 ± 120.13	16.82

**Figure 3:**
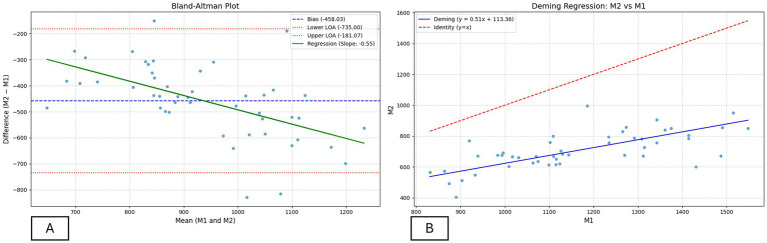
Agreement analysis between BMD assessment methods (M1 and M2). **(A)** Bland–Altman plot showing the mean bias (−458.0 HU), limits of agreement (−735.0 to −181.1 HU), and regression of the difference versus mean (slope = −0.55), indicating proportional bias. **(B)** Deming regression between M1 and M2 (slope = 0.51; 95% CI: 0.34–0.68), showing a positive relationship but with M1 overestimating BMD relative to M2.

Pearson’s correlation (Table 3) between BMD values obtained by the two methods indicated a significant positive linear association (r = 0.6822). The Bland–Altman plot (Figure 3A) showed a mean bias of −458.0, with 95% limits of agreement ranging from −735.0 to −181.1. According to Table 3, regression of the difference against the mean presented a significant slope (β = −0.55; p < 0.001), a low Lin’s concordance correlation coefficient (CCC = 0.12; 95% CI: 0.07–0.17), associated with a reduced bias correction factor (Cb = 0.16), as well as Deming regression (Figure 3B) with an intercept of 113.4 (95% CI: −76.9 to 303.5) and a slope of −0.55 (95% CI: 0.34–0.68).

**Table 3:** Agreement indicators between the two methods (M1 vs. M2).

Agreement indicator	Value	95% CI	*p*-value
Pearson’s correlation (*r*)	0.682	-	<0.001
CCC (Lin’s Concordance Correlation Coefficient)	0.120	0.07–0.17	<0.001
Mean bias (M2-M1)	−458.0	-	-
Limits of agreement	−735.0; −181.1	-	-
Bias correction factor (Cb)	0.16	-	-
Deming regression (α)	113.4	−76.9; 303.5	-
Deming regression (β)	0.51	0.34; 0.68	-

### 3.2 Associations with bone variables and eggshell traits

**Figure 4:**
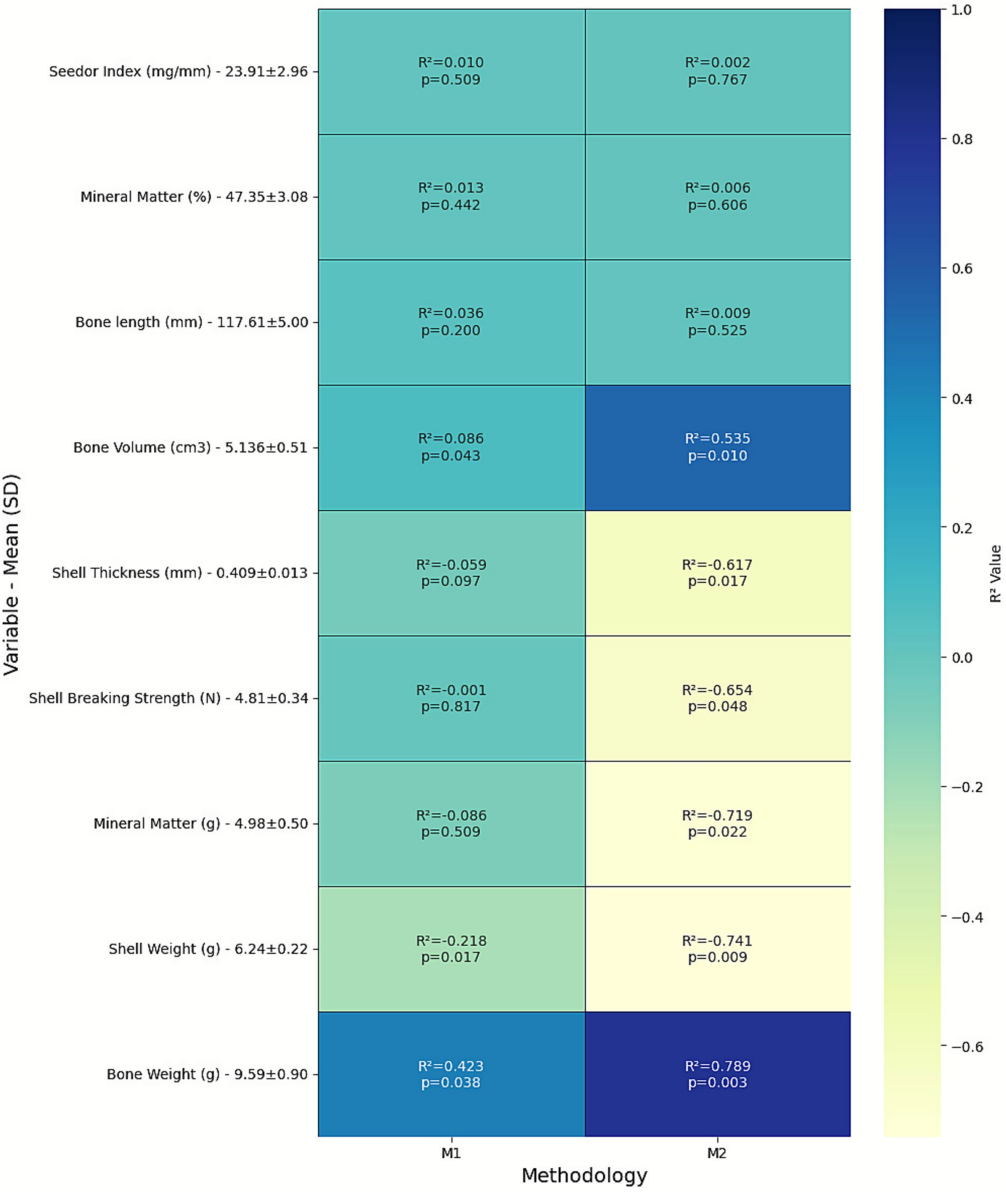
Heatmap of determination coefficients (*R*^2^) and *p*-values showing the relationships between BMD (M1 and M2) and bone/eggshell variables. Darker blue tones indicate higher positive *R*^2^ values, while lighter yellow tones indicate negative correlations.

Most bone variables showed weak correlations with bone mineral density (BMD) (Figure 4). Bone length was not significantly associated with either methodology (M1: R^2^ = 0.036, *p* = 0.200; M2: R^2^ = 0.009, *p* = 0.525), nor were the percentage of mineral matter and the Seedor index, both presenting R^2^ values close to zero and non-significant (M1: R^2^ = 0.013, *p* = 0.442 and R^2^ = 0.010, *p* = 0.509, respectively; M2: R^2^ = 0.006, *p* = 0.606 and R^2^ = 0.002, *p* = 0.767, respectively).

In contrast, variables related to bone composition showed distinct correlation patterns with BMD, particularly for M2. Bone weight (R^2^ = 0.789, *p* = 0.003) and bone volume (R^2^ = 0.535, *p* = 0.010) exhibited strong positive correlations with BMD, indicating that denser bones were also heavier and larger in volume.

However, mineral matter in grams showed a negative correlation with BMD in both methods, being significant only for M2 (R^2^ = −0.719, *p* = 0.022) and non-significant for M1 (R^2^ = −0.086, *p* = 0.509).

For M1, overall correlations were weaker, with bone weight (R^2^ = 0.423, *p* = 0.038) and bone volume (R^2^ = 0.086, *p* = 0.043) showing only mild associations with BMD.

Regarding eggshell traits, negative correlations were observed between BMD and eggshell characteristics, indicating that hens with higher bone density tended to produce lighter shells. Shell weight was negatively correlated with BMD (M2: R^2^ = −0.741, *p* = 0.009; M1: R^2^ = −0.218, *p* = 0.017). Shell thickness (M2: R^2^ = −0.617, *p* = 0.017) and breaking strength (M2: R^2^ = −0.654, *p* = 0.048) also showed significant negative relationships, while associations for M1 remained weak and non-significant (shell thickness: R^2^ = −0.059, *p* = 0.097; shell strength: R^2^ = −0.001, *p* = 0.817).

## 4 Discussion

### 4.1 Methodological agreement and bias

The results demonstrate differences between methods for assessing bone mineral density (BMD) in tibiae of laying hens. Although Pearson’s correlation was positive, the other indicators revealed low agreement between methods, preventing their use as equivalent approaches. The concordance coefficient and Bland–Altman plot highlighted a systematic bias with wide limits of agreement, in which M2 consistently yielded lower values than M1. Deming regression indicated proportional bias (slope = 0.51), with greater differences observed at higher BMD values.

The analysis of methodological agreement revealed that M1 consistently produced higher BMD values (1152.49 mgHA/cm^3^) than M2 (711.22 mgHA/cm^3^), with a mean difference of approximately 441 mgHA/cm^3^. This variation reflects a systematic overestimation associated with the region-based approach used in M1.

Previous studies evaluating different genetic lines and ages of laying hens have reported tibial BMD values ranging from 400 to 800 mgHA/cm^3^ (8, 11), demonstrating that physiological bone density in laying hens can vary substantially depending on genotype, age, and analytical methodology.

It is worth noting that these studies employed different imaging techniques, such as micro-computed tomography (micro-CT) and peripheral quantitative computed tomography (pQCT), which may also contribute to variability in the reported values. Therefore, the difference observed between M1 and M2 in the present study likely reflects methodological factors related to image segmentation and sampling area, rather than intrinsic biological variation.

Regional variations in tibial bone mineral density (BMD) observed in this study reflect the complex structural and functional remodeling that occurs during the laying period. The medial region showed the highest BMD values in both CT-based methods, likely due to its predominantly cortical composition. This compact architecture provides mechanical support and resistance to bending forces, allowing the midshaft to serve as the tibia’s primary load-bearing zone.

Similar patterns have been consistently reported in laying hens and other avian species, where cortical thickening in the mid-diaphysis contributes to structural stability (32).

In contrast, the proximal region showed signs of cortical and trabecular bone loss, with reduced mineral density and decreased trabecular connectivity, collectively weakening its structural integrity. The distal region showed a comparable pattern, characterized by resorption of cortical and trabecular bone and the formation of medullary bone. Although medullary bone functions as a vital calcium reservoir for eggshell formation, it is less dense and mechanically weaker than structural bone, thereby increasing overall skeletal fragility (33).

Taken together, these findings indicate that bone remodeling in the tibia during the laying phase involves substantial loss of structural bone in both the proximal and distal regions, accompanied by formation of medullary bone that facilitates calcium mobilization but compromises bone strength. This process leads to cortical thinning, trabecular rarefaction, and increased medullary deposition, generating an imbalance between structural maintenance and mineral utilization that predisposes hens to long-term skeletal fragility and fractures.

These discrepancies stem from the intrinsic characteristics of each method. Method 1 (M1) prioritizes regions of higher radiological density, thus overestimating mean BMD by disregarding less dense areas of cortical bone. In contrast, Method 2 (M2) provides a more comprehensive estimate by considering the entire bone area, reducing errors by avoiding reliance on manually selected points.

Harrison et al. (25) and Jones et al. (34) employed methodologies that segment the cortical and trabecular bone area to obtain more consistent estimates of bone quality. However, those studies did not account for regional variations along diaphysis, which may compromise anatomical representativeness. Conversely, Santos et al. (24) and Ribeiro et al. (35) analyzed multiple regions of diaphysis but restricted measurements to small, manually selected cortical areas. Such an approach tends to overestimate BMD by emphasizing denser points while disregarding structural variations related to cortical thickness and the presence of medullary bone, factors directly linked to bone strength and quality (17).

The present study combines both strategies by applying whole-area segmentation at standardized levels of diaphysis, thus offering a more representative assessment and minimizing selection bias.

### 4.2 Correlation with bone parameters and eggshell quality

Another important finding was the superiority of M2 in reflecting bone variables and eggshell traits. Regression analyses showed that M2 had significant associations with mineral deposition indicators such as bone weight, mineral matter (g), and bone volume, whereas M1 exhibited weak associations. Bone densitometry in laying hens, particularly using QCT, has been shown to correlate strongly with bone mineral content, including ash and calcium, and moderately to strongly with bone weight and volume (19). Among the evaluated methods, M2 demonstrated greater sensitivity in capturing these differences, more accurately reflecting both mineral content and structural variation, likely due to its broader segmentation of cortical and medullary areas.

The ash-to-volume ratio reflects structural integrity, and BMD is influenced by dietary calcium, being higher in birds with adequate levels, especially in the tibia (36, 37). The relationship between mineral density and bone characteristics varies with age and laying cycle, particularly in long bones, where density fluctuates during egg production (38).

Bone length and the Seedor index showed limited predictive capacity for BMD evaluation by CT due to the low variability of these measures. Previous studies indicate that bone growth and mineralization are not uniform, and that microarchitectural organization plays a more decisive role in determining mineral density (39).

Regarding the relationship between BMD and eggshell traits, both the magnitude and direction of the correlations observed in this study are biologically meaningful. Consistent with previous reports, our results showed negative correlations between BMD and eggshell characteristics such as shell thickness, weight, and breaking strength, particularly for M2. This pattern indicates that hens with higher skeletal mineralization mobilize less calcium for shell formation, resulting in shells that are generally lighter and potentially less resistant. Such an inverse association reflects a metabolic trade-off between maintaining bone mineral reserves and supporting continuous eggshell calcification. These findings reinforce that skeletal integrity and eggshell quality are physiologically interdependent outcomes regulated by calcium homeostasis during the laying cycle (40, 41).

In the present study, M2 proved more efficient at detecting these variations, suggesting that BMD assessment may serve as an indirect predictor of eggshell quality.

From a practical perspective, the choice of methodology directly influences the accuracy of studies on skeletal health and egg quality. The whole-area segmentation approach demonstrated greater robustness, reproducibility, and correlation with functional parameters, making it more suitable for experimental evaluations and with potential for *in vivo* applications in longitudinal studies.

The association observed between M2-based BMD, which relates to bone composition and eggshell traits, highlights the potential of quantitative CT for large-scale, non-invasive monitoring of skeletal health in laying hens. Such correlations suggest that M2 can be effectively used in vivo studies, allowing for translational assessment of bone integrity throughout the production cycle. Additionally, advances in deep learning algorithms have enabled automatic segmentation of specific bones from whole-body CT or radiographic images, as recently demonstrated for keel bone detection in laying hens (42).

These developments open new perspectives for integrating imaging-based phenotyping into genetic selection programs and for enhancing welfare-oriented breeding strategies focused on bone strength and resilience.

Although keel bone fractures represent the most prevalent skeletal issue in commercial laying hens, particularly across cage and non-cage systems (43), tibial bone density remains a heritable and reliable indicator of overall skeletal robustness. Accurate quantification of tibial BMD, as demonstrated in this study, can contribute to genomic selection programs aimed at improving bone strength and reducing fracture susceptibility (44).

Enhancing tibial mineralization may also benefit the structural integrity of the keel bone, as birds with stronger long bones tend to exhibit better load distribution and reduced fracture risk (45). These findings reinforce the value of CT-based approaches not only for physiological assessment but also for long-term breeding strategies focused on bone health and animal welfare.

However, some limitations should be noted: the relatively small number of birds, the use of a single commercial strain at a relatively young age, and the restriction of analysis to the tibia. Moreover, M1 relies on manual ROI selection, whereas M2 requires strict standardization of density thresholds. Therefore, results should be interpreted with caution and validated in different strains, ages, and housing conditions. In addition, ex vivo BMD measurements can be affected by factors such as sample temperature and image reconstruction artifacts, which may alter HU calibration and introduce minor variability in quantitative results.

## 5 Conclusion

In summary, the results indicate that point-based analysis of cortical regions provides consistent but limited information on mineralization. In contrast, whole-area cortical assessment more comprehensively captures variations related to bone quality and eggshell traits. Therefore, complete cortical segmentation demonstrates greater validity and practical applicability and is recommended for studies investigating the interaction between bone metabolism and eggshell quality in laying hens.

## Data Availability

The original contributions presented in the study are included in the article/supplementary material, further inquiries can be directed to the corresponding author.
